# MiR-629 promotes human pancreatic cancer progression by targeting FOXO3

**DOI:** 10.1038/cddis.2017.525

**Published:** 2017-10-26

**Authors:** Haijiao Yan, Qing Li, Jun Wu, Wenwei Hu, Jingting Jiang, Liangrong Shi, Xin Yang, Danxia Zhu, Mei Ji, Changping Wu

**Affiliations:** 1 Department of Oncology, The Third Affiliated Hospital of Soochow University, Changzhou 213003, P.R. China; 2 Department of Pathology, The Third Affiliated Hospital of Soochow University, Changzhou 213003, P.R. China; 3 Department of Tumor Biological Treatment, The Third Affiliated Hospital of Soochow University, Changzhou 213003, P.R. China

## Abstract

The FOXO signaling pathway has been reported to have an important role in human cancer. Expression of miR-629 was markedly upregulated in pancreatic cancer and negatively correlated with FOXO3. Therefore, exploring the regulatory mechanism of miR-629 and FOXO3 signaling may provide valuable clinical targets for pancreatic cancer therapy. In the current study, we found that overexpressing and inhibiting miR-629, respectively, enhanced and reduced the cell proliferation and metastasis of pancreatic cancer cells *in vitro* and *in vivo* compared with parental cells or cells transfected with a control vector. Furthermore, we found that miR-629 negatively regulated FOXO3 protein expression and decreased the activity of a luciferase reporter construct containing the FOXO3 3′-untranslated region. These results show that miR-629 regulates FOXO3 at the posttranscriptional level, resulting in enhanced cell proliferation and invasion of pancreatic carcinoma. Furthermore, we found that overexpressing miR-629 enhanced, while inhibiting miR-629 reduced, the stem cell-like phenotype of pancreatic cancer cells *in vitro*. A functional polymorphism at miR-629-binding site in the 3′-UTR of FOXO3 gene confers a decreased risk of progression in pancreatic carcinoma. Furthermore, these findings suggest that miR-629 has a vital role in promoting the development of pancreatic cancer and may represent a novel prognostic biomarker and therapeutic target.

Pancreatic cancer is a highly lethal malignancy with a median survival of <6 months. Pancreatic ductal adenocarcinoma (PDAC) is the most common form, which accounts for >90% of all the pancreatic cancer cases.^1^ Currently, studies have shown 63 types of genetic mutations in pancreatic cancer, and these mutations are mainly present in 12 signaling pathways. However, there are challenges in targeting these pathways for pancreatic therapy, and thus to explore and develop other critical approaches for pancreatic cancer treatment is still important and necessary.

MicroRNAs are a uniquely stable set of ubiquitously expressed small noncoding RNAs that belong to a class of small non-coding RNAs approximately 19–25 nucleotides in length and are able to bind complementary sequences in the 3′-untranslated regions (3′-UTR) of various target mRNAs to promote degradation or translational repression.^2, 3^ In cancer, miRNAs modulate stemness, EMT, expression of tumor-suppressor genes and oncogenes, and many other essential pathways that phenotypically affect cancer cells, such as drug resistance, tumor growth, invasion, and metastasis.^4, 5, 6, 7, 8^ In the present study, we found that expression of miR-629 was upregulated in pancreatic cancer tissues and cell lines. MiR-629 regulated FOXO3 protein expression negatively at the posttranscriptional level and enhanced cell proliferation and invasion of pancreatic carcinoma *in vivo* and *in vitro*. Our data provide strong evidence for the potential use of miR-629 as a novel approach to treat human pancreatic cancer.

## Result

### MiR-629 expression was negatively correlated with FOXO3 in pancreatic cancer

**Figure 1:**
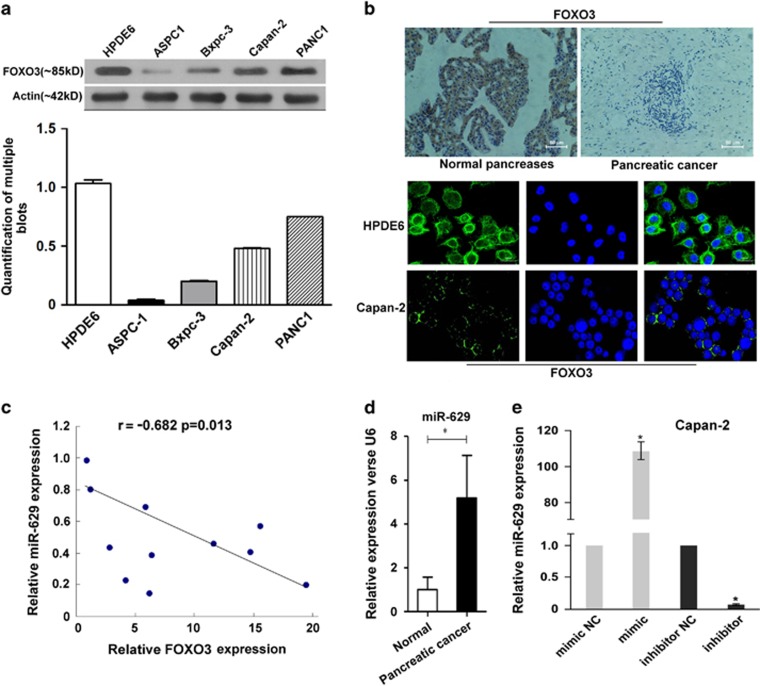
The expression of miR-629 and FOXO3 in pancreatic carcinoma cells was negatively correlated. (**a**) Western blotting of FOXO3 in a human immortalized pancreatic ductal epithelial cell line HPDE6 and four pancreatic cancer cell lines (ASPC-1, Bxpc-3, Capan-2, and PANC-1) and the quantification of multiple blots; (**b**) Immunohistochemistry detection of FOXO3 in paraffin-embedded tissue sections and immunofluorescence staining of FOXO3 in Capan-2 (× 200). (**c**) Real-time RT-PCR detection of miR-629 and FOXO3 in pancreatic carcinoma tissues and their correlation. (**d**) Mean value of relative miR-629 expression by real-time RT-PCR in the 11 matched pancreatic carcinoma and normal pancreatic tissue (*P<0.01). (**e**) Real-time RT-PCR detection of miR-629 after transfection with miR-629 mimic or miR-629 inhibitor and the control sequence in Capan-2 cells. The results are normalized to U6B expression and are presented as relative miR-629 expression (*P<0.01)

To investigate the FOXO3 expression in pancreatic cancer, western blotting was performed on a human immortalized pancreatic ductal epithelial cell line HPDE6 and a panel of four pancreatic cancer cell lines (ASPC1, Bxpc-3, Capan-2, and PANC1) Our data showed that the expression of FOXO3 protein was strongly positive in human immortalized pancreatic ductal epithelial cell compared with that in pancreatic cancer cell lines (Figure 1a). Furthermore, immunohistochemistry and for paraffin-embedded tissue sections and immunofluorescence for the cell lines showed that FOXO3 was localized in the cytoplasm of cells, and the expression of FOXO3 in normal pancreas tissue and HPDE6 was stronger than in pancreatic cancer cells (Figure 1b). It should be noted that FOXO3 expression was inversely correlated with miR-629 in pancreatic carcinoma tissues according to real-time PCR (Figure 1c). According to the analysis of miR-629 by real-time PCR for the 11 matched pancreatic carcinoma and normal pancreatic tissue, our data showed that the expression level of miR-629 was higher in pancreatic carcinoma tissues than that in the normal pancreatic tissues (*P*<0.01; Figure 1d). In addition, miR-629 could be upregulated or downregulated in the Capan-2 cell line when transfected with the miR-629 mimic or inhibitor (*P*<0.01; Figure 1e).

### Inducing proliferation effects of miR-629 on pancreatic cancer cells *in vitro*

**Figure 2:**
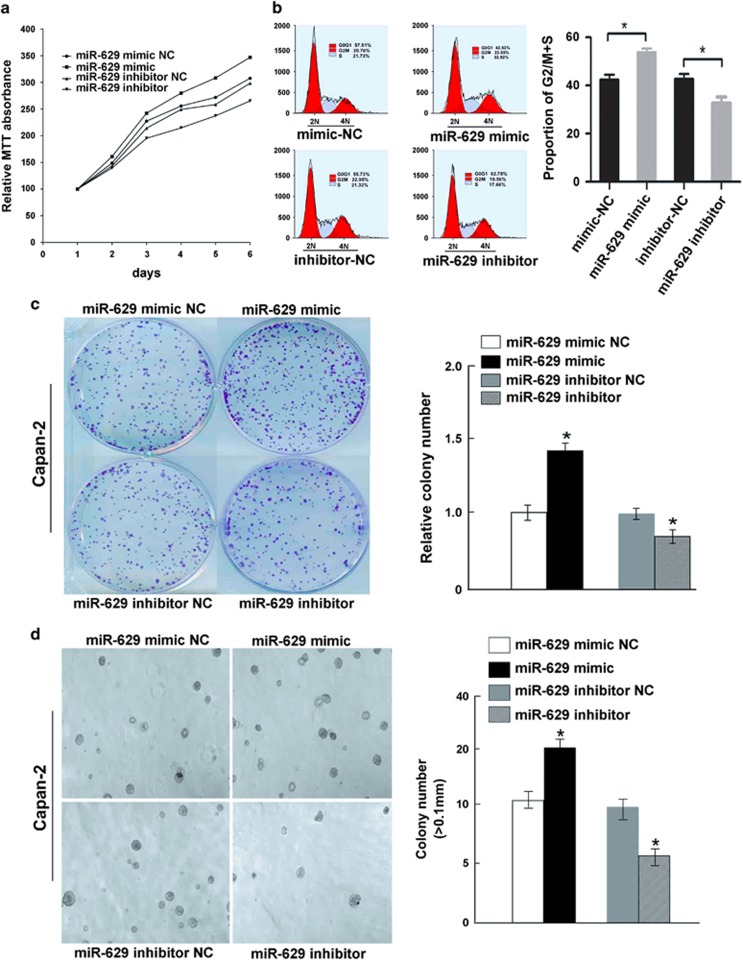
Antiproliferative effects of miR-629 on pancreatic cancer cells *in vitro*. (**a**) MTT experiment. Cell viability was significantly increased in cells with overexpression of miR-629 compared with control cells in a time-dependent manner. Values represent means±S.D. (*P<0.01). (**b**) Cell cycle assay. Cell cycle analysis indicated an increased population of cells at the G2/M+S phase by miR-629 overexpression. The experiment was repeated three times (*P<0.05). (**c**) Clone-formation assay. The effect of miR-629 overexpression on cell proliferation was evaluated with crystal violet staining. Values represent the means±S.D. of three experiments from three independent tests (*P<0.05). (**d**) Analysis of sphere formation. miR-629 accelerated sphere formation of pancreatic carcinoma cells (*P<0.05)

3-(4,5-Dimethylthiazol-2-yl)-2, 5-diphenyltetrazolium bromide (MTT) assays were performed with Capan-2 cells in which miR-629 was upregulated or downregulated, as well as the control groups. The cell growth curves showed that the growth rate of tumor cells was significantly increased by overexpression of miR-692 in a time-dependent manner, but the reverse result was obtained in miR-692 downregulated cells (Figure 2a). The maximum increase rate for Capan-2 was 16.68±1.33% and the inhibitory rate was 10.75±1.56% on day 6 (*P*<0.05). There was no significant difference between the control cells (*P*>0.05; Figure 2a). Cell cycle distribution showed that overexpressed miR-629 was significantly different from that of the control groups, with a higher proportion of cells within G2/M+S phase (57.66±0.3% *versus* 43.33±0.2%, *P*<0.01; Figure 2b), but the G0/G1 phase population was significantly increased in the cells transfected with miR-629 inhibitors (63.28±0.4% *versus* 52.55±0.5%, *P*<0.01; Figure 2b). We also evaluated the effect of ectopic expression of miR-629 on cell proliferation through clonogenic assay. Compared with the control groups after 10 days, proliferation of Capan-2 cells transfected with miR-629 mimic was increased to 46.68±1.27% (*P*<0.01; Figure 2c), while the maximum inhibitory rate of miR-629 inhibitors was 31.21±1.32% (*P*<0.01; Figure 2c). A tumor sphere-formation assay showed that miR-629-transduced cells formed a larger number of spheres with increased diameter compared with vector control cells (*P*<0.01; Figure 2d).

### Promotion of cell growth by FOXO3 siRNA in miR-629 downregulated Capan-2 cells

**Figure 3:**
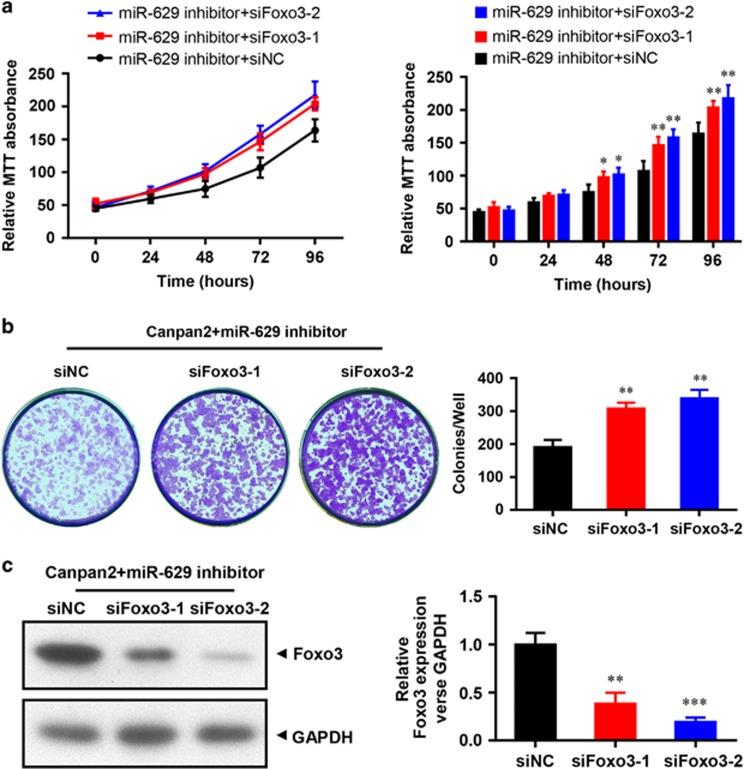
Promotion of cell growth by FOXO3 siRNA in miR-629 downregulated Capan-2 cells. (**a**) MTT experiment. Cell viability was significantly increased in a time-dependent manner when siFOXO3-1 or siFOXO3-2 were cotransfected with miR-629 inhibitor in Capan-2 cells. Values represent means±SD (*P<0.05, **P<0.01). (**b**) Clone-formation assay. The effect of siFOXO3-1 or siFOXO3-2 on cell proliferation in miR-629-inhibited Capan-2 cells was evaluated with crystal violet staining. Values represent the means±S.D. of three experiments from three independent tests (**P<0.01). (**c**) Downregulation of FOXO3 protein expression detected by western blotting analysis and the quantification of multiple blots (**P<0.01)

In our study, the data showed that RNAi directed against FOXO3 significantly increased the growth rate of tumor cells in a time-dependent manner, and the highest proliferation rates were 28.02±2.85 and 35.36±2.67% for siFOXO3-1 and siFOXO3-2, respectively, on day 8 (*P*<0.01, Figure 3a). We also detected the effect of siFOXO3-1 and siFOXO3-2 on cell proliferation in miR-629-downregulated cells through clone-formation assay. Compared with the control group after 10 days, proliferation rates of siFOXO3-1 and siFOXO3-2 groups increased to 30.15±0.57% and 33.66±0.78% (*P*<0.01; Figure 3b), respectively. Western blotting showed that FOXO3 protein was reduced in the siFOXO3-1 and siFOXO3-2 groups in miR-629-inhibited Capan-2 cells (*P*<0.01; Figure 3c).

### MiR-629 overexpression promotes CSC-like traits in pancreatic cancer cells

**Figure 4:**
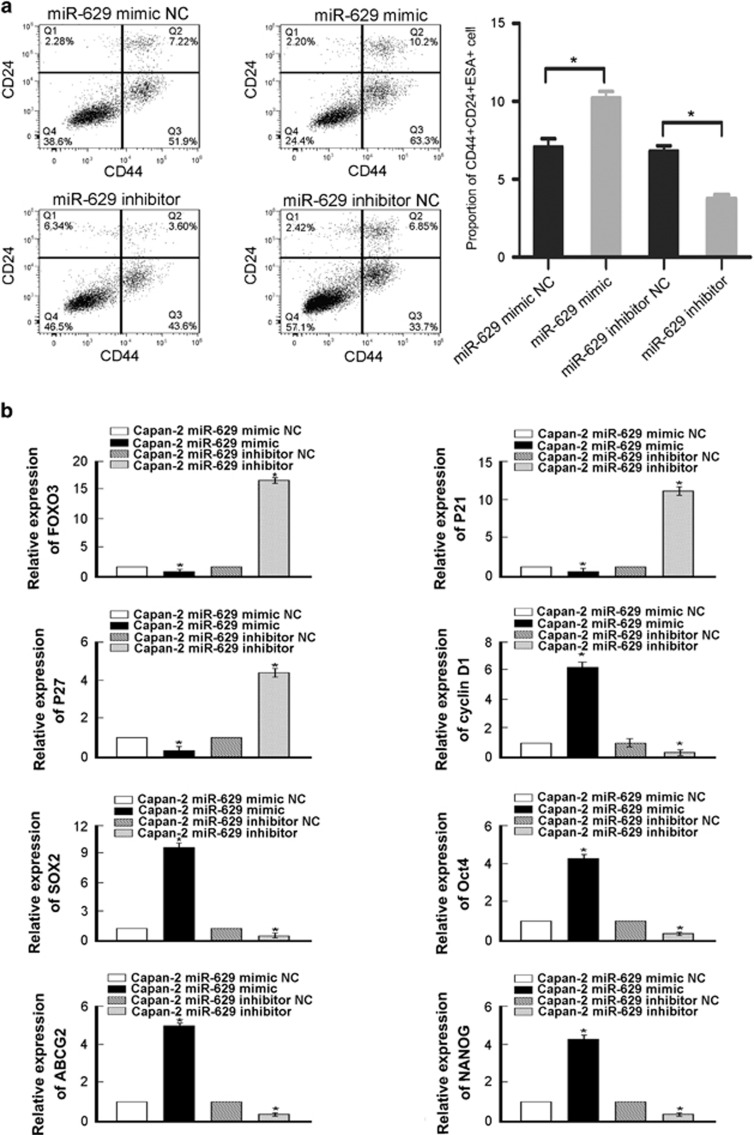
MiR-629 inhibition promotes CSC-like traits in pancreatic cancer cells. (**a**) Distribution of CD44+CD24+SEA+ cells using a flow cytometric analysis. The experiment was repeated three times (*P<0.05). (**b**) Real-time RT-PCR analysis of mRNA expression that miR-629 overexpression significantly upregulated the mRNA expression levels of stem cell markers, including CyclinD1, ABCG2, OCT4, SOX2, and NANOG, but downregulated the mRNA expression levels of multiple pluripotency factors, including FOXO3, p21, and p27, in the indicated cells. Transcript levels were normalized to GAPDH expression. Error bars represent the mean±S.D. of three independent experiments (*P<0.05)

To understand the biological role of miR-629 in pancreatic cancer progression, miR-629 mimic was transduced into the Capan-2 pancreatic cancer cell line, and we found that miR-629 overexpression significantly increased the population of CD44+CD24+ESA+ cells compared with the control cells (Figure 4a). Nevertheless, populations of cells that were positive for the pancreatic CSC marker CD44+CD24+ESA+ cells were dramatically decreased in miR-629 inhibitor-transduced cells compared with the control group (Figure 4a). Furthermore, we found that miR-629 overexpression significantly upregulated the mRNA expression levels of multiple pluripotency factors, including CyclinD1, ABCG2, OCT4, SOX2, and NANOG (Figure 4b). In addition, we found that miR-629 overexpression significantly downregulated the mRNA expression levels of multiple pluripotency factors, including FOXO3, p21, and p27 (Figure 4b). Thus our experiments further indicate that miR-629 might act as a CSC inducer. Collectively, our data suggest that miR-629 overexpression might promote a stem cell-like phenotype in pancreatic cancer cells.

### Overexpression of miR-629 promotes metastasis of pancreatic cancer cells *in vitro* and *in vitro*

**Figure 5:**
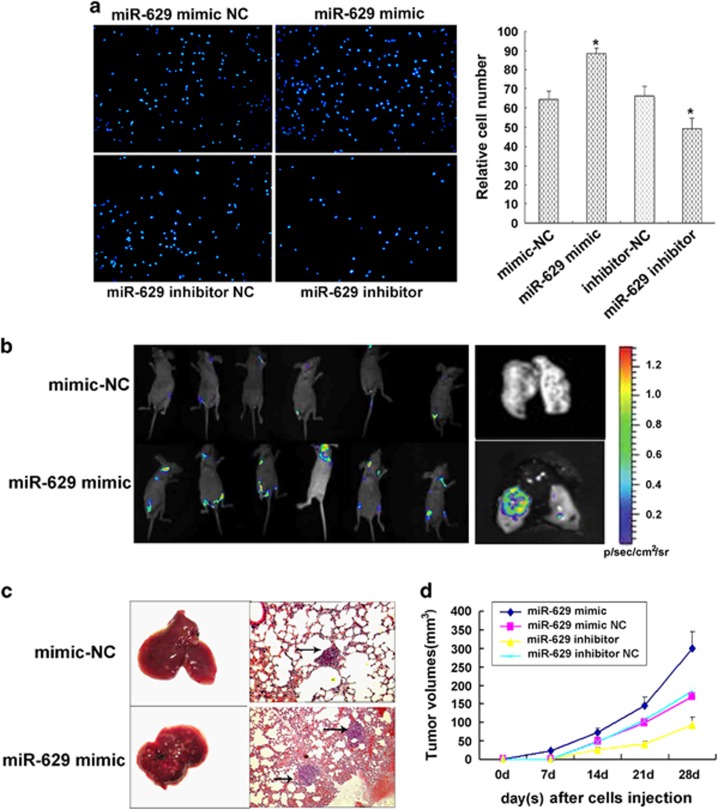
Overexpression of miR-629 promotes metastasis of pancreatic cancer cells *in vitro* and *in vivo*. (**a**) Transwell migration assays and Matrigel invasion assays showed inhibition of cellular invasion by overexpression of miR-629. Columns represent the means±S.D. *P<0.05. (**b**) The antitumor effect of miR-629 overexpression on pancreatic carcinoma xenografts. Two weeks postvector injection, tumor volume was imaged by Fluc bioluminescence imaging. (**c**) Macroscopic view of lung specimens of mice: there were many small nodules on the surface of the lung in miR-629 mimic group but it was smooth in the control group. Pulmonary metastatic tumor H&E staining (× 200): the metastasis nodi are indicated by arrows. (**d**) Tumor cells were injected subcutaneously into nude mice. The volume of each tumor was measured each week. Values represent means±SD. *P<0.05. Mice were killed after 4 weeks, and tumors were resected and weighed. Values represent means±S.D. *P<0.05

An *in vitro* invasion system was used to evaluate the effect of miR-629 overexpression on the invasion of pancreatic carcinoma cells. Compared with control group, miR-629 mimic resulted in a significant enhancement in migratory and invasive capacity in Capan-2 cells. The migration and invasion rate of cells transduced with miR-629 mimic was increased to 38.25±1.25% and 29.22±1.36%, respectively (*P*<0.05; Figure 5a). Furthermore, our data indicated that the migration and invasion rate of cells transfceted with miR-629 inhibitor was reduced to 27.35±1.22% and 28.65±1.55%, respectively (*P*<0.05; Figure 5a). Two weeks postvector injection, tumor volume was imaged by Fluc bioluminescence imaging. The Fluc signal significantly increased in miR-629 mimic group on day 14 (Figure 5b), whereas the Fluc signal in the control group continuously decreased over time. The volume of tumors resulting from injection of cells with overexpression of miR-629 were significantly bigger compared with those from the control group (*P*<0.05, Figure 5d), and the volume of the miR-629-inhibitor group were significantly less (*P*<0.05, Figure 5d). We used caudalis vena injection of tumor cells to evaluate the effects of miR-629 overexpression on tumor metastasis. The mice were killed 28 days after tumor cell injection when they had become moribund. Seeding of these pancreatic tumors by lung metastasis-derived cells was observed in 6/6 (100%) of mice. The cells that overexpressed miR-629 produced a large number of lung metastatic nodules (*P*<0.05; Figure 5c). Lung metastasis nodus was analyzed by histopathological examination with hematoxylin and eosin (H&E) staining (Figure 5c).

### MiR-629 levels correlate with p21^cip1^, Cyclin D1, β-catenin, p-AKT, and p-GSK3*β* protein expression in pancreatic cancer cell line Capan-2

**Figure 6:**
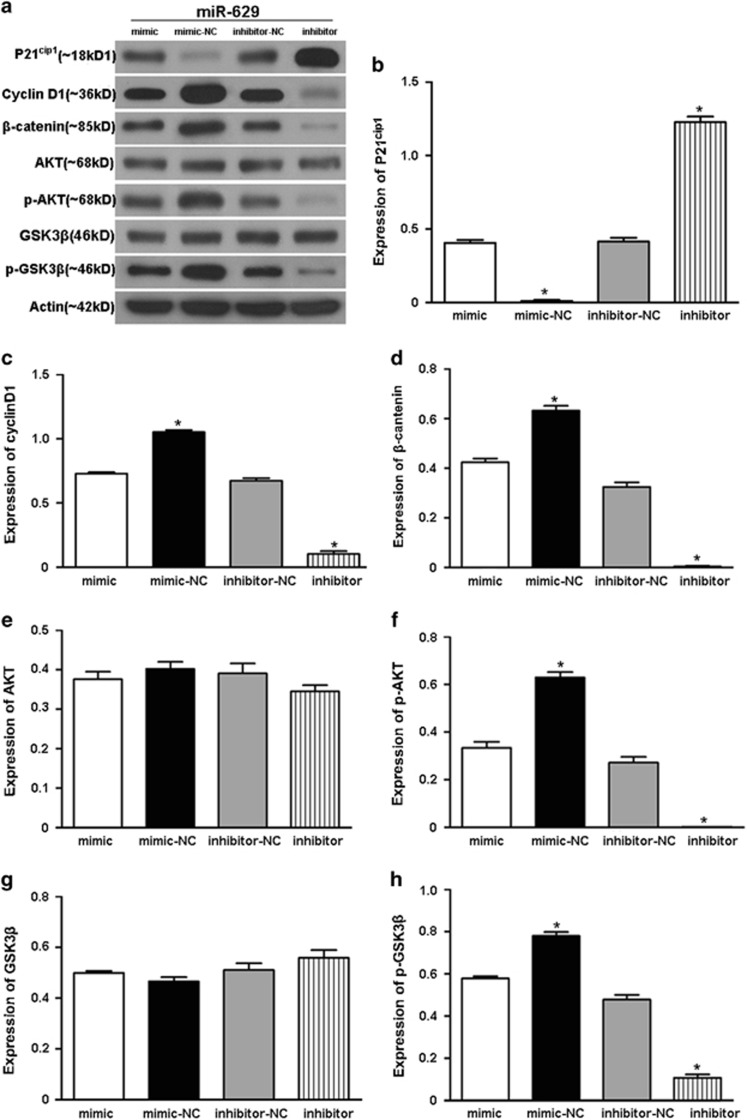
Involved gene that were correlated with miR-629. (**a**) Total lysates obtained from Capan-2 cells transfected with miR-629 mimic or inhibitor were separated by SDS-PAGE and immunoblotted with the indicated antibodies. Images are representative of three independent experiments. (**b**–**h**) Quantification of the multiple blots. Data are presented as the means±S.D. of three independent experiments (*P<0.05)

Activation of the phosphoinositide 3-kinase (PI3K)/AKT pathway is correlated with poor patient prognosis.^9^ GSK-3*β* is another molecule that controls cell cycle and apoptosis in the Akt signaling pathway. GSK-3*β* also interacts with *β*-catenin to regulate cell proliferation through Wnt signaling. If Akt is inactivated, GSK-3*β* can phosphorylate and inactivate *β*-catenin, leading to the inhibition of cell survival stimulated by Wnt signaling.^10, 11, 12^ In our results, western blotting analysis revealed that Cyclin D1, *β*-catenin, p-AKT, and p-GSK3*β* expression was enhanced in miR-629-overexpressed Capan-2 cells (Figures 6a, c, d, f, and h), while the opposite results were observed in the miR-629-inhibited Capan-2 cells. AKT and GSK3*β* were not changed in either group (Figures 6a, e, and g). However, p21^cip1^ was downregulated in the miR-629-overexpression group and upregulated in the miR-629-inhibitor group (Figures 6a and b).

### MiR-629 regulates FOXO3 by targeting the FOXO3 3′-UTR

**Figure 7:**
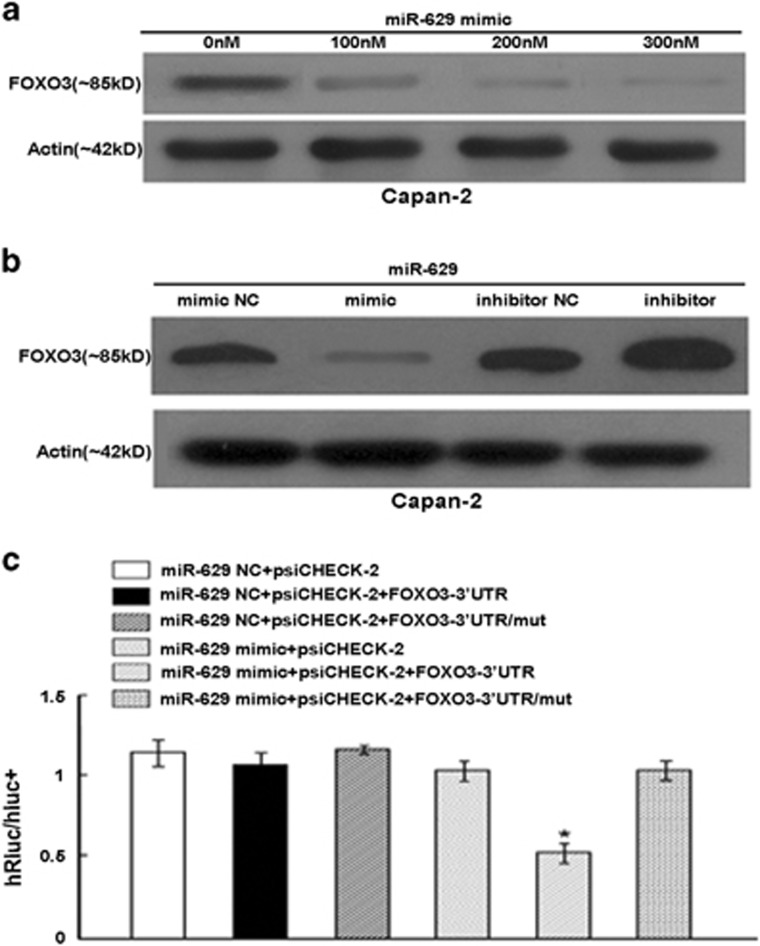
MiR-629 regulates FOXO3 by targeting the FOXO3 3′-UTR. (**a**)The FOXO3 protein expression after transfection with 100–300 nM miR-629 mimic. (**b**) Western blotting analysis of FOXO3 expression in Capan-2 cells transfected with miR-629 mimic or inhibitor compared with control cells. Protein expression was quantified and normalized to *β*-actin. (**c**) Luciferase activity of the wild-type FOXO3 3′-UTR reporter gene and mutants cotransfected with miR-629 mimics or scramble. Columns represent the means±S.E. (*P<0.01)

Our results showed that the FOXO3 protein level was decreased in a dose-dependent manner when Capan-2 cells were transfected with 100–300 nM concentrations of miR-629 mimic (Figure 7a). And the FOXO3 protein level was significantly reduced in cells transfected with miR-629 mimic and increased in cells transfected with miR-629 inhibitor compared with those from the control groups (Figure 7b). To determine whether the 3′-UTR of FOXO3 mRNA is a functional target of miR-629, a reporter plasmid driven by the SV40 basal promoter harboring the two miR-629-binding sites in the FOXO3 3′-UTR was constructed, as well as mutant reporters (Figure 7c). Both the wild-type and the mutant reporters were introduced into the Capan-2 cell lines. We found that ectopic expression of miR-629 inhibited the luciferase activity of the reporter vector containing wild-type FOXO3 3′-UTR, FOXO3-M1, (*P*<0.01; Figure 7c). Taken together, these data suggest that both microRNA-binding sites on the FOXO3 3′-UTR are functional and that FOXO3 is one of the direct targets of miR-629.

## Discussion

Numerous studies have demonstrated that microRNAs are clinically correlated with cancer proliferation, invasion, metastasis, and chemo-resistance, which leads to high morbidity and mortality among patients,^13, 14, 15, 16, 17^ and miRNAs are correlated with the mechanism by which pancreatic CSCs maintain a stem cell-like phenotype.^18^ Herein we found that overexpression of miR-629 in pancreatic cancer enhanced, whereas suppression of miR-629 inhibited, the CSC-like phenotypes. Consistently, *in vivo* studies revealed that fortifying miR-629 significantly promoted the tumorigenicity and lung metastasis of pancreatic cancer cells. These findings uncover a novel mechanism for pancreatic cancer proliferation and metastasis and suggest a potential therapeutic effect of miR-629. We proposed that regulated expression of miR-629 may disrupt cell cycle control, in turn, promoting cell proliferation and metastasis, and consequently facilitating the development of pancreatic cancer. Moreover, we identified that miR-629 negatively regulates the oncogene FOXO3 by binding to the 3′-UTR of FOXO3 mRNA in pancreatic carcinoma cells. Taken together, we suggest that overexpression of miR-629 is a potential therapy targeting proliferation and invasion pathways in pancreatic carcinoma.

FOXO3 is a key mediator of Akt and extracellular signal-regulated kinase (ERK)-mediated CSC regulation. The same group also identified FOXO3 as a key mediator of the ERK- and Akt-mediated regulation of glioblastoma stem cells.^19^ And FOXO3 is activated in colorectal CSCs and non-CSCs upon inhibition of the Akt and ERK pathways by ONC201/TIC10.^20^ Our study demonstrate here that simultaneously targeting an epigenetic mediator and an epigenetic modulator, by dual inhibiting OCT4 and AKT, can have significantly improved efficacies over single treatment in suppressing the propagation of CSCs as well as the entire bulk of differentiated cancer cells. Self-renewal and pluripotency are indispensable features in the definition of stem cell populations and some transcription factors such as Sox2, Nanog and Oct4 are key regulators of these processes.^21, 22^ Oct-3/4, Sox-2, and Nanog3 are pluripotent markers that are commonly expressed by the embryonic stem cells. Oct-4 forms a ternary complex with Sox-2, which is known as Oct4–Sox-2 complex.^23^ Similar to Oct-4 and Sox-2, Nanog is also a key regulator essential for embryonic stem cell pluripotency and self-renewal.^24^ Also OCT4 induced activation of TCL1, AKT, and ABCG2 in the process of inducing chemoresistance, suggesting a new potential pathway.^25^ Its expression is quickly downregulated upon stem cell differentiation. ABCG-2 is expressed by the immature hematopoietic stem cells and its expression is sharply downregulated upon differentiation.^26^ It is also abundantly expressed in the placenta, which has a role in the efflux of the xenobiotic components. Hence the non-significant change in the expression of ABCG-2 after serial passage could be due to the balancing role that ABCG-2 has in the regulation of the stem cells’ differentiation, apoptosis and growth. In the current study, Sox2, Oct-4, ABCG2, and NANOG were simultaneously upregulated in miR-629-overexpressed pancreatic cancer cells, and the expression of p21 and p27 was inversely correlated with the expression of miR-629 in pancreatic cancer cells. These data suggest a novel regulatory mechanism for Sox2, Oct-4, ABCG2, and NANOG and indicate that increased expression of p-AKT, p-GSK3*β*, and *β*-catenin could enhance cancer cell stemness.

FOXO3 is the central mediator of the pro-proliferative PI3K/AKT signaling pathway in which AKT phosphorylation causes inactivation, nuclear exclusion, and subsequent degradation of FOXO3.^27, 28, 29^ Other kinases such as I*κ*-B kinase17 and ERK^30^ can also phosphorylate and downregulate FOXO3 activity in a similar manner.^31^ FOXO3 can also antagonize the functions of FOXM1, which is a potent oncogene that has a central role in promoting cell proliferation, migration, invasion, angiogenesis, stem cell renewal, and DNA damage repair processes, which contribute to cancer initiation, progression, and drug resistance.^31, 32^ FOXO3 is also a molecular target of multiple clinically available or potential anticancer therapeutics, and therefore, its deregulation could culminate in drug resistance.^33, 34^ Past studies have reported that FOXO3 is primarily regulated by multiple kinases that can phosphorylate FOXO3, which subsequently leads to nuclear exclusion and ubiquitination in the cytoplasm.^35^ In this study, we found that overexpression of miR-629 inhibited FOXO3 protein expression by directly binding to its 3′-UTR, which resulted in increasing proliferation and metastasis in pancreatic cancer cells.

In summary, we found that suppression of miR-629 enhanced, whereas overexpression of miR-629 inhibited FOXO3 through directly targeting 3′-UTR. Furthermore, p21^cip1^ inversely correlated, while p-AKT, p-GSK3*β*, and nuclear *β*-catenin positively expression correlated with the expression of miR-629 in pancreatic cancer clinical specimen. Moreover, the expression of miR-629 was positively correlated with tumorigenesis both *in vitro* and *in vivo*. These findings suggest miR-629 as a potential therapeutic target. Based on our current results and previous findings, we here propose a model of a regulation of proliferation signaling pathway involving miR-629.

## Materials and methods

### Tissue samples and cell lines

The 11 pancreatic carcinoma tissues used in this study were obtained from patients in The Third Affiliated Hospital of Soochow University from 2010 to 2016. The samples were obtained with patients’ informed consent and were histologically confirmed. All tissue samples were derived from untreated patients undergoing surgery, fixed by formalin immediately, and embedded in paraffin. The PDAC cell line Capan-2 was obtained from the American Type Culture Collection (ATCC, Manassas, VA, USA) and maintained in DMEM supplemented with 10% fetal bovine serum (Sigma-Aldrich, St. Louis, MO, USA), 100 units/ml penicillin, and 100 mg/ml streptomycin. All the cells were cultured in a humidified 37 °C incubator with 5% CO_2_. Cells were tested regularly for mycoplasma (using the new MycoProbe Mycoplasma Detection Kit from R&D Systems, Minneapolis, MN, USA). All cell lines were grown under identical conditions.

### Immunocytochemistry

Tumor cells were harvested by trypsinization and seeded onto poly-L-lysine-treated glass slides for 24 h. The cells were then fixed for 5 min in methanol at −20 °C, and immunocytochemistry was performed as reported previously.^3^

### miRNA and siFOXO3 transfection

Cells in exponential phase were plated in 60-mm plates at 1 × 10^6^ cells/plate, cultured for 16 h, and then transfected with the miR-629-mimic, miR-629 inhibitor, or scramble using Lipofectamine 2000 (Invitrogen Life Technologies, Carlsbad, CA, USA) according to the manufacturer’s protocol. The cell groups were named miR-629-mimic-NC, miR-629-mimic, miR-629-inhibitor-NC, and miR-629-inhibitor. The effects of miR-629-mimic and miR-629-inhibitor on the expression of FOXO3 and other proteins was examined 48 h after transfection. According to the human FOXO3 gene sequence provided by GenBank (NM-001168), the tow FOXO3 siRNA were designed and synthesized as the following sequences: siFOXO3-1: 5′-UAUACGGGAAGCUAGAGCUCCGCUG-3′ and antisense, 5′-CAGCGGAGCUCUAGCUUCCCGUAUA-3′, and siFOXO3-2: 5′-AUUAUAGGCUGACGUGGCAUUCCUA-3′ and antisense, 5′-CCUGCCCGGU GAACUCUCCUAUAAU-3′.

### Cell proliferation and assessment of clonogenicity

Twenty-four hours after transfection with miR-629 mimic, inhibitor, and scramble, cells were plated in triplicate in 96-well plates at 3 × 10^3^ viable cells/well overnight. Cell viability was determined by MTT assay. The absorbance at 490 nm was read on a spectrophotometer (Shimadzu, Columbia, USA). For the clone-formation assay, the tumor cells were seeded at low density (5 × 10^3^ cells per 10-cm^2^ plate) and incubated for 10 days. Colony formation and growth were visualized with crystal violet staining. After the wells were photographed, the dye was solubilized with methanol, and the optical density at 590 nm was measured with an ELISA reader. The surviving fraction was calculated relative to the control cells.

### Real-time PCR

A real-time PCR-based method was used to quantify the expression levels of miRNA using a protocol described previously.^36^ The prepared RT product (1 ml) was used as the PCR template. Each PCR reaction contained YBR Master Mix (Applied Biosystems, Foster City, CA, USA), 200 nM miRNA-specific forward primer, and 200 nM universal reverse primer. All reactions were performed in duplicate on an ABI Prism 7500 fast real-time PCR system (Applied Biosystems). The conditions for quantitative PCR were 95 °C for 10 min, followed by 40 cycles of 95 °C for 15 s and 63 °C for 32 s. Expression of U6 small nuclear RNA was used as an endogenous control for data normalization. The threshold cycle (Ct) is defined as the cycle number at which the fluorescence intensity change crosses the average background level of the fluorescence signal. In the initial screening studies, the normalized miRNA level was defined by the equation (39-Ct after normalization to the internal control) with global median normalization before further analysis. For analysis of individual miRNA expression, the value of each Ct was first normalized to U6 small nuclear RNA (snRNA), and then the normalized miRNA level was defined by the equation (39-Ct after normalization to the expression of U6 snRNA).

### Cell cycle analysis using flow cytometry

Transfected Capan-2 cells and untransfected cells were seeded in six-well plates. When the cells were ~80% confluent, The experiment was performed as reported previously.^3^ The cell cycle distribution was analyzed using flow cytometry (BD Biosciences, Franklin Lakes, NJ, USA). The experiment was repeated three times.

### Flow cytometric analysis

Cells were dissociated with trypsin and suspended at 1 × 10^6^ cells/ml in DMEM containing 2% FBS and then incubated at 37 °C for 30 min with or without 100 *μ*M verapamil (Sigma-Aldrich) to inhibit ATP-binding cassette (ABC) transporters. The cells were subsequently incubated for 90 min at 37 °C with 5 *μ*g/mL Hoechst 33342 (Sigma-Aldrich). Lastly, cells were incubated on ice for 10 min and washed with ice-cold phosphate-buffered saline (PBS) prior to flow cytometric analysis. Flow cytometric analysis or flow cytometric cell sorting was conducted using fluorescein isothiocyanate (FITC)-conjugated monoclonal mouse anti-human CD44, CD24, and ESA antibodies (Miltenyi Biotec GmbH, Bergisch Gladbach, Germany). Samples were analyzed and sorted on BD FACSCanto II and FACSAria I instruments, respectively (BD Biosciences), and the data were analyzed using the FlowJo software (Tree Star Inc., San Carlos, CA, USA). The experiment was repeated three times.

### Migration and invasion assay *in vitro*

Invasion assays were performed using a specialized invasion chamber (BD Biosciences). And experiment was performed as reported previously.^3^ The migration rate and invasion rate were quantified by counting the migratory cells in six random fields. The experiment was repeated three times.

### *In vitro* Fluc bioluminescence imaging

Mice were anesthetized with isoflurane and then received an injection of D-luciferin (150 mg/kg body weight diluted in PBS). Fifteen minutes later, the mice were placed in the imaging chamber, and photo counts were acquired for 1–5 min by the optical imaging system (IVIS 50 Imaging System; Xenogen Technology, Hopkinton, MA, USA). Signal intensity quantification and analysis were performed using the Living Image Software (version 2.50; Xenogen Technology) provided by the manufacturer. The bioluminescent signal was recorded as maximum photons/s/centimeter^2^/steradian (photon/s/cm^2^/sr), represented in a pseudo-color photo count manner and superimposed on the photographic image, displaying both bioluminescence intensity and the anatomy of the mice.

### Western blotting analysis

After 48 h of transfection with miR-629-mimic and miR-629-inhibitor (400 nM), Capan-2 cells were harvested, and then 80 pg of total protein extract was separated on 10% SDS-PAGE gels and transferred to PVDF membranes (BD Biosciences). The membrane was probed with monoclonal anti-FOXO3 and anti-p21 antibodies (1 : 500, Santa Cruz Biotechnology, Santa Cruz, CA, USA), as well as with anti-Cyclin D1 (1 : 500, Cell Signaling, Shanghai, China), anti-AKT, anti-phospho-AKT, anti-GSK3*β*, anti-phospho-GSK3*β*, and anti-*β*-actin (1 : 1000, Sigma–Aldrich, St.Louis, MO) antibodies. The membrane was further probed with peroxidase-conjugated secondary antibodies at optimized concentrations, and the protein bands were visualized using enhanced chemiluminescence (Amersham Pharmacia Corp., Piscataway, NJ, USA).

### 3′-Luciferase reporter assay

The 3′-UTR of FOXO3 mRNA containing the miR-629-binding site was amplified by PCR, and the product was inserted into the XbaI site of a PsiCHECK2 vector (Promega, Madison, WI, USA). The primers used for the amplification were Homo-FOXO3-3′UTR-F-497(XhoI) (5′-CCGCTCGAGAGGACA GAACCGTGCATAGG-3′) and Homo-FOXO3-3′UTR-R-497(NotI) (5′-ATAAGAAT GCGGCCGCGCCTCTCACTCATACACTTC-3′). A mutant with a deletion of 5 bp from the fully complementary site was also generated using a Quik-Change II Site-Directed Mutagenesis Kit (Stratagene, La Jolla, CA, USA). Wild-type and mutant inserts were confirmed by sequencing. Twenty-four hours before transfection, Capan-2 cells were plated at 1.5 × 10^5^ cells/well in 24-well plates, and then the cells were transfected with 800 ng of PsiCHECK2-FOXO3-3′-UTR or PsiCHECK2-mutFOXO3-3′-UTR. Forty-eight hours after transfection, luciferase activity was assayed using a Dual-Luciferase Reporter Assay System (Promega).

### Statistical analysis

Each experiment was repeated at least three times. The results were calculated using the SPSS 16.0 software (SPSS Inc., Chicago, IL, USA), and analysis of variance was used to determine significant differences among the groups. Differences were considered statistically significant when *P*<0.05.

## Publisher’s Note

Springer Nature remains neutral with regard to jurisdictional claims in published maps and institutional affiliations.
